# Revisiting the policy ecology framework for implementation of evidence-based practices in mental health settings

**DOI:** 10.1186/s13012-023-01309-9

**Published:** 2023-11-07

**Authors:** Whitney K. Wortham, Aaron H. Rodwin, Jonathan Purtle, Michelle R. Munson, Ramesh Raghavan

**Affiliations:** 1 Silver School of Social Work, New York University, 1 Washington Square North, New York, NY 10003 USA; 2 Department of Public Health Policy & Management, Global Center for Implementation Science, School of Global Public Health, New York University, 708 Broadway, New York, NY 10003 USA

**Keywords:** Mental health, Evidence-based practice, Mental health parity, Consumer involvement, De-implementation, Adoption, Sustainment

## Abstract

**Background:**

Over the past three decades, policy actors and actions have been highly influential in supporting the implementation of evidence-based practices (EBPs) in mental health settings. An early examination of these actions resulted in the Policy Ecology Framework (PEF), which was originally developed as a tactical primer for state and local mental health regulators in the field of child mental health. However, the policy landscape for implementation has evolved significantly since the original PEF was published. An interrogation of the strategies originally proposed in the PEF is necessary to provide an updated menu of strategies to improve our understanding of the mechanisms of policy action and promote system improvement.

**Objectives:**

This paper builds upon the original PEF to address changes in the policy landscape for the implementation of mental health EBPs between 2009 and 2022. We review the current state of policy strategies that support the implementation of EBPs in mental health care and outline key areas for policy-oriented implementation research. Our review identifies policy strategies at federal, state, agency, and organizational levels, and highlights developments in the social context in which EBPs are implemented. Furthermore, our review is organized around some key changes that occurred across each PEF domain that span organizational, agency, political, and social contexts along with subdomains within each area.

**Discussion:**

We present an updated menu of policy strategies to support the implementation of EBPs in mental health settings. This updated menu of strategies considers the broad range of conceptual developments and changes in the policy landscape. These developments have occurred across the organizational, agency, political, and social contexts and are important for policymakers to consider in the context of supporting the implementation of EBPs.

**Summary:**

The updated PEF expands and enhances the specification of policy levers currently available, and identifies policy targets that are underdeveloped (e.g., de-implementation and sustainment) but are becoming visible opportunities for policy to support system improvement. The updated PEF clarifies current policy efforts within the field of implementation science in health to conceptualize and better operationalize the role of policy in the implementation of EBPs.

**Supplementary Information:**

The online version contains supplementary material available at 10.1186/s13012-023-01309-9.

Contributions to the literature

- We provide an update of the Policy Ecology Framework (PEF), which has been widely used to examine a variety of policy implementation actions, and identify and examine the utilization of policy strategies in contemporary practice settings.
- We identify conceptual and policy changes across organizational, agency, political, and social contexts in the area of the implementation of evidence-based practices in mental health.
- We describe how PEF can be used to select policy strategies that support adoption, sustainment, and de-implementation decisions that align with calls from the field for expedient investigation into policy’s role in implementation efforts.

## Background

How might policymakers best support the implementation of evidence-based practices (EBPs) in mental health care? The potential answers to this question have evolved over time as the conceptualization of what exactly constitutes a policy intervention, its appropriate targets, and their intended and unintended effects, have become better understood. Here, we think of both policy and non-policy implementation strategies as actions; yet, the distinction between these strategies is contingent upon the actors, the context, and the level of intended impact [1]. Over the past two decades, the thrust of policy efforts has shifted from mandating the adoption of specific practices to supporting more general implementation milieus. For example, this includes a shift from focusing on specific interventions to an approach that builds provider capacity, and from creating specific policy enablers for practices to supporting broader dissemination efforts [2]. As policy-focused work within implementation science has evolved [3–5], underdeveloped policy targets such as de-implementation and sustainment, are emerging as opportunities for policy support [6]. Inventorying these targets in the current policy environment, and documenting the changes in policy actions that have occurred since the last inventory was published in 2008 as the Policy Ecology Framework [7], is the goal of this manuscript.

In this paper, we conceptualize policies as *strategies* policymakers can deploy to support the adoption, implementation, spread, and sustainment of EBPs. This should not, however, be confused with “policy implementation,” which refers to the implementation of legislative and regulatory rules and procedures [8, 9]. EBPs—not policies—are “the thing” of focus per Curran’s terminology [10]. Per Purtle et al.’s typology of ways to approach policy in implementation science, our conceptualization is consistent with the category of “policy as strategy to use” [1].

## Overview of the Policy Ecology Framework

The Policy Ecology Framework (PEF) [7] was developed as a tactical primer for state and local mental health regulators in the field of child mental health. Its original purpose was to present a menu of strategies (i.e., “policy levers”) for policymakers as they partnered with treatment developers and mental health center administrators to improve care for children exposed to traumatic stress. Like other determinant frameworks in the field of implementation science [11], PEF posits that there is a broad ecology surrounding the delivery of EBPs (beyond individual clinician and provider organization factors) that contributes to the success of implementation. The ecology consists of several contexts—organizational, agency, political, and social—defined in Table 1.

**Table 1:** Summary of shifts in policy strategies since the publication of the original PEF in 2008

Level in the policy ecology	Context description	Key changes
Organizational context	• Clinical settings within which EBPs are delivered	• Original PEF refers to as a service delivery organization, i.e., community mental health center • Affordable Care Act changed the organizational landscape: ◦ Fourteen states have adopted Medicaid ACOs or ACO-like entities ◦ Sixteen states are considering adoption of Episodes of care programs ◦ Value-based purchasing implemented in 48 jurisdictions • Prior authorization is largely extinct • Rise of novel marketplaces (Children’s Service Funds)
Agency context	• Local or state bodies that oversee or influence this organizational activity	• Changed environment for contracting and bidding towards outcomes-based purchasing
		• Increased consumer involvement
		• Expanded loan forgiveness programs (largely due to ACA) expanded workforce
Political context	• All legislative and advocacy efforts that support the implementation of EBPs	• Both the Mental Health Parity and Addiction Equity Act and the ACA were passed after the publication of PEF
		• Increased emphasis on racial justice, structural stigma, and institutional racism in regulation
		• Increase in legislation directed at specific evidence-based practices (e.g., Family First Prevention Services Act)
Social context	• Cultural and structural factors that shape access to EBPs	• Increasing awareness of structural stigma (not just self-stigma), and institutionalized racism • Increasing efforts to meaningfully include individuals with lived experience in the research and dissemination process to ensure that outputs and activities involve coproduction

The PEF has been used to examine a variety of policy actions across the USA. These include local policy efforts, such as Philadelphia’s attempts to transform its behavioral health system [12], implementing trauma interventions in a citywide group of schools [13], and evaluating the implementation of an intervention for homeless persons [14]. Examples of state-level actions that have been assessed using PEF include Maryland’s efforts to support EBPs within their Medicaid Health Home Program [15], and Minnesota’s efforts to enhance equity for cultural and ethnic minority persons through its Cultural and Ethnic Minority Infrastructure Grant program [16]. Collectively, these studies suggest policymakers have utilized a subset of the PEF’s policy strategies to drive systems change.

## Aims and objectives

This paper aims to identify and enumerate additional policy levers and to retire unused policy levers so that policymakers, advocates, and implementation science practitioners have a more contemporary set of policy tools to guide efforts toward EBP implementation improvement. More specifically, we aim to provide an updated menu of strategies and expand the scope of the original PEF to address updates across organizational, agency, political, and social context landscapes. We focused on updating two main areas: (1) identifying emergent policy strategies at federal, state, agency, and organizational levels, and highlighting developments in the social context in which mental health EBPs are implemented; and (2) identifying changes in policy strategies and their impact on the implementation of mental health EBPs between 2009 and 2022. To accomplish this, we reviewed policy strategies that have been identified as potentially supporting the implementation of EBPs in mental health care and outline sunrise areas for policy-oriented implementation research (Additional file 1). Here, our emphasis is on public policy as well as what is referred to as “small p” policy (i.e., healthcare systems and organizational policies) [17].

Since the 2008 PEF, a range of developments in the policy landscape have impacted the implementation of EBPs in mental health. First, some policy levers have been subject to further conceptual development (e.g., bidding and contracting [18–21]). Second, federal legislation has produced a set of novel policy levers (e.g., the rise of Accountable Care Organizations [ACOs] [22], and expansions of value-based purchasing). Third, there has been increasing international interest and deployment of policy strategies to drive health system reform efforts, especially in Asia [23]. Most importantly, the PEF did not emphasize that policy action enhances adoption, while organizational strategies largely support implementation [24, 25]. These changes in the policy landscape require a revisiting of the PEF strategies. Furthermore, our interrogation and enumeration of these newer strategies furthers our understanding of the mechanisms of policy action, thereby furthering a policy “science of how” [26].

## Discussion

**Fig. 1:**
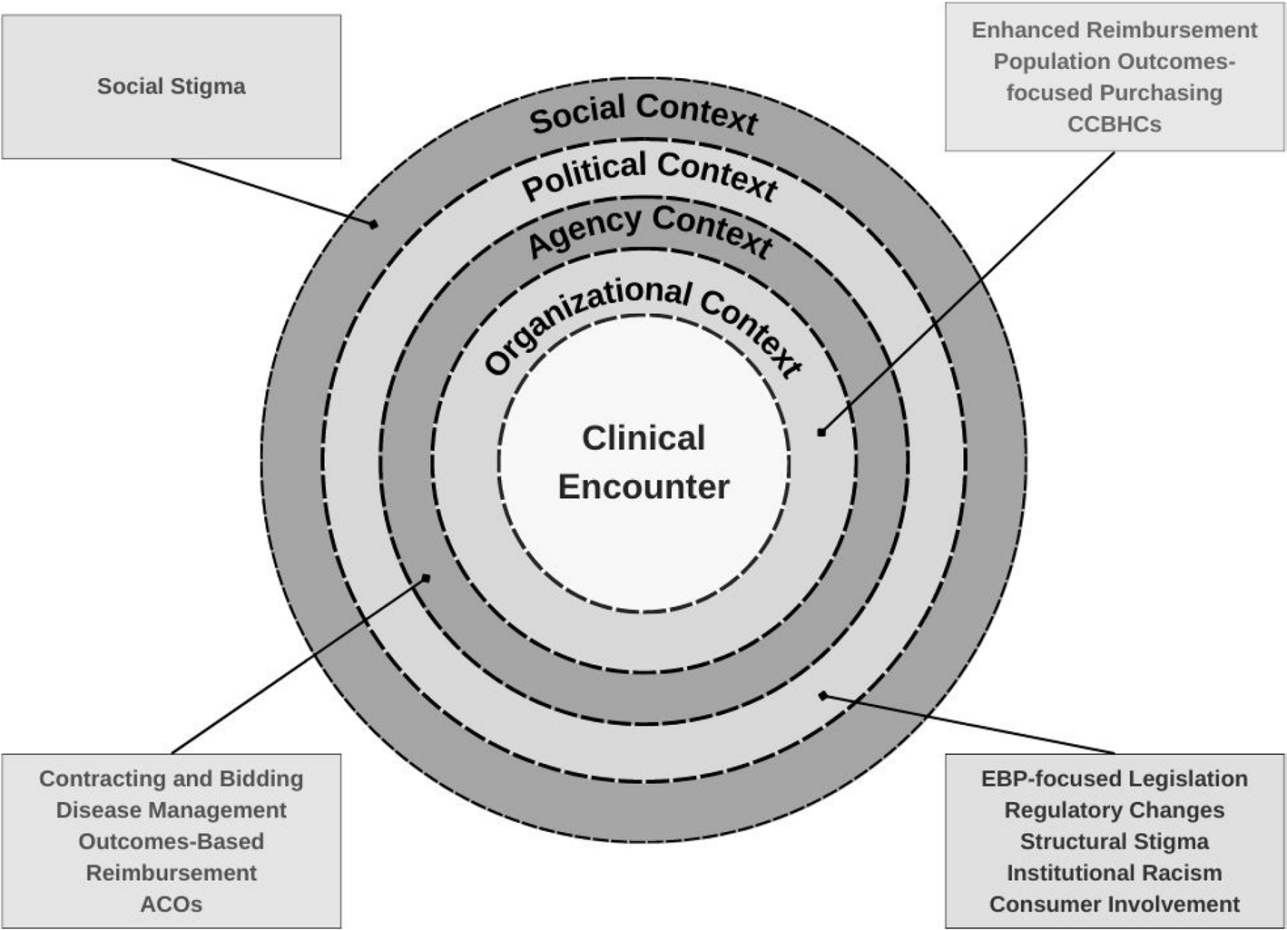
Policy Ecology Framework 2.0

Figure 1 presents the revised PEF that reflects substantive updates to key domains which are described in detail below. Dashed lines are used to represent the interplay and reciprocity across levels in the ecology as the boundaries are permeable.

### Changes in the organizational context

Since the original PEF, several changes have occurred in the organizational context surrounding the delivery of mental health services including (1) enhanced reimbursement; (2) value-based purchasing, and (3) novel organizational arrangements. These changes are largely linked to the passage of the Patient Protection and Affordable Care Act (ACA).

#### Enhanced reimbursement

EBPs in mental health are typically expensive to gain expertise in, and to deploy and sustain [27]. The past decade has seen considerable expansions of the necessity to support the added costs of implementation [28]. Many interventions are receiving enhanced reimbursement in various states [29]. Enhanced rates are especially important for interventions that are complex and expensive, and increased rates are part of the reason for the success of multisystemic therapy implementation in New Mexico [30]. Enhanced reimbursement can also be provider-focused (e.g., expanding the range of individuals who can be reimbursed for providing mental health interventions, such as peer specialists) [31]. These enhanced reimbursement models work in a generally uniform way—they are designed for specific treatments (e.g. trauma-focused cognitive behavior therapy), usually work with a dedicated billing code providers can use, and restrict the use of these codes to providers who have met certain state requirements. These enforcements are typically backed up by audit flags.

#### Population outcomes-focused purchasing

One way to deploy enhanced reimbursement is via billing codes for specific interventions. Another emerging way to increase EBP delivery is to enhance reimbursements for condition-specific or population-specific care and require the use of EBPs within them. For example, California provides enhanced reimbursement rates for care for persons with serious mental illness and/or addictions, and for those who are houseless [32]. The purpose of funding is not to support the delivery of specific EBPs, but to reduce disparities—a population-level outcome—among disadvantaged populations [32]. Another population-specific initiative is OhioRISE (Resilience through Integrated Systems and Excellence). Designed for youth with complex behavioral health needs, the program incorporates both value-based and incentive-based (i.e., both outcome-based and volume-based in this context) financial supports that are higher than standard Medicaid reimbursements [33]. This type of fiscal model is a type of “bundled” reimbursement with an emphasis on achieving broader population-focused outcomes. These reimbursement strategies differ from narrower value-based purchasing programs (detailed below). Currently, there is substantial design complexity and programmatic heterogeneity in these types of programs; yet, the core idea of increasing reimbursement for specific service packages is an increasingly common way to support EBP implementation.

#### Novel organizational arrangements

Another way that policies can promote EBP deployment is to reengineer the organization that delivers them. In 2014, the Protecting Access to Medicare Act created a demonstration program to establish and evaluate certified community behavioral health clinics (CCBHCs). As of 2022, there were 450 CCBHCs operating in the USA [34]. CCBHCs receive enhanced Medicaid reimbursement rates for services. In exchange, they provide nine defined types of services (e.g., 24-h mobile crisis mental health care teams), and are required to deliver an array of services, including EBPs, that are not only focused on specific treatments but also cover service integration and treatment planning [35, 36]. CCBHCs are one type of organizational arrangements that have emerged in recent years. Below, we discuss Accountable Care Organizations ([ACOs; groups of healthcare providers who coordinate care, take responsibility for total cost and quality of care, and, in return, receive a portion of the savings they achieve) [37].

These novel arrangements reside in parallel to the kinds of arrangements identified in the original PEF—purchasing cooperatives, service delivery cooperatives, public–private partnerships, health plan-sponsored provider networks, provider-supported or -directed care organizations, and a similar array of structural and institutional mechanisms to deliver health care. While these organizational arrangements are usually driven by financial arrangements, the extent to which they represent a better implementation model remains to be seen.

### Changes in the agency context

Since the original PEF, several changes have occurred in the agency context that relates to the following areas: (1) expansion of agency-level tools; (2) contracting and bidding; (3) disease management; (4) prior authorization; (5) outcomes-based reimbursement; and (6) ACOs.

#### Expansions of agency-level tools

Originally focused on service delivery agencies (e.g., state departments of mental health) and financing agencies (e.g., state Medicaid departments), the PEF aligned with calls from implementation scientists to cultivate a “tailored selection” of strategies specific to the goals, barriers, and contextual demands of an implementation effort [12, 38–40]. Now, owing to federal and state policy actions, regulatory agencies (child welfare or mental health departments) have greatly increased their implementation-focused activities. They have been aided in their efforts by advisory/evaluative bodies (e.g., the Washington State Institute for Public Policy), novel financing mechanisms (e.g., new sources of Medicaid funding for home and community-based services [41]), quasi-public funding bodies (e.g., Children’s Service Funds), and expanded roles of accreditation bodies (e.g., the Council on Accreditation that accredits human services providers). A succeeding section on changes in the political landscape summarizes key legislative efforts driving the adoption and implementation of EBPs.

Though states vary significantly, policymakers apportion resources to support EBP infrastructure and enact regulations that dictate which services are available and reimbursable under state Medicaid plans. Subsequently, these actions work together to influence EBP implementation in practice [20]. In a recent study, several factors (e.g., per capita income, controlling political party, Medicaid expansion) predicted the level of state fiscal investments in adopting EBPs in public mental health systems [42]. By contrast, modifiable factors (e.g., interagency collaboration and investment in research centers) were more predictive of state policies supportive of EBPs. State per capita debt and direct state operation of services (versus contracting for services) predicted both child and adult EBP adoption [43]. 

Regulatory changes have provided new, or reformulated, tools for policymakers, including (1) defining levels of evidence; (2) funding mandates/funding targets; (3) codifying laws that aid implementation; (4) establishing state inventories that classify programs by evidence of effectiveness; and (5) increased oversight and monitoring of EBPs [43, 44]. Policymakers in Oregon, Washington, Utah, Minnesota, and Connecticut, for example, have made legislative changes to support the implementation of EBPs—allocating at least 50 percent of purchasing dollars towards EBPs [43]. Additionally, 39 states have defined one level of evidence at minimum, 49 states have created an inventory of funded EBPs and have employed targeted funding to support those effective programs, and 33 states have created laws to sustain support for the implementation of these programs [43, 44] 

#### Contracting and bidding 

The PEF assumed that specialized contracting and bidding occupied an exclusively intra-organizational locus. Currently, drawing from key constructs of the Exploration, Preparation, Implementation, Sustainment (EPIS) framework, these policy actions are better understood as “bridging factors.” [19, 45–48]. System-wide efforts to implement EBPs have grown largely due to the deployment of fiscal policies (provided for by the ACA, discussed below), and contracting procedures and performance-based contracting have emerged as key tools in shaping the implementation context of agencies and local service agencies [49, 50]. 

Contracts often operate as an ‘on–off’ switch for the implementation and sustainment of EBPs within community mental health systems [51, 52]. They have begun to dictate performance targets, compensation for service delivery, and determine the level of funding—directly impacting the agency [18]. Within county-based health systems, contracts tether county agencies and private, non-profit agencies to fill service gaps [18, 53]. Contracts are key elements of a multi-level fiscal support mechanism increasingly seen across the USA that braid contracting, incentives, fees for service, and grants [38, 50]. Contracts explicate the expectations of organizations to deliver EBPs and, in turn, communicate system-level support of agencies and their service environments. 

Between agencies and service systems, there is a bi-directional flow of information in which contracting serves as the conduit for this information [19, 50, 54]. For example, Walker and colleagues [55] highlight the role of contracting agencies in their case study of a state-level, EBP service delivery tracking system in Washington state. Reporting EBP use per session and, thus, the number of sessions in an agency and healthcare system as a contract requirement increased awareness and motivation amongst contracted agencies and increased transparency and social pressure to implement EBPs. Service system-level decision-makers influence the type of care delivered by agencies and utilize contracts to specify to whom and by whom this care is delivered. 

#### The evolution of disease management

The original PEF identified state-level efforts to improve the quality of healthcare by applying a disease management framework. Many of those elements (e.g., identifying high-risk patients, matching interventions to patient needs), have changed following the passage of the ACA. Section 2703 of the ACA instituted an option for states to receive a 90% enhanced Federal Medical Assistance Percentage to establish health homes to connect Medicaid beneficiaries with chronic conditions to coordinated healthcare services [56]. Health homes offer care coordination services with core elements being patient education, monitoring and appointment reminders, and linkages to behavior modification programs [57]. Once connected to a health home, people have access to a team of providers including those who deliver mental health EBPs and substance use services, which is particularly important in rural and remote communities [58]. CMS (Centers for Medicare and Medicaid Services) determined eligibility for health homes and provided protection from exclusion of the benefits for people with both Medicare and Medicaid, but allowed state-level regulation of how health home services were distributed geographically. An explicit goal of the health home program–holistically treating two or more chronic conditions and a serious and persistent mental health condition—is a current iteration of disease management strategies. In these ways, the ACA has modified the venues and structures of disease management programs, while retaining disease management as a model for implementing best practices.

#### Prior authorization

Prior authorization (PA)—originally intended as a measure of cost containment to control pharmaceutical expenditures—has persisted, albeit much reduced and modified, in the last decade. The advent of mental health parity brought to bear the utility of PA for payors with respect to managing resource utilization (i.e., inpatient psychiatric hospitalization and intensive outpatient services where EBPs are largely utilized) [59]. Overall, PA remains functional as a policy lever to moderate access to mental health EBPs, particularly for managed care enrollees, as PA determines the scope and duration of benefits [60, 61]. There has been persistent criticism of prior authorization from the provider community, as many providers cited concerns over administrative burden, lack of transparency in determinations, and hampered access to timely care; all of which have contributed to federal efforts to streamline the PA process [62].

#### Outcomes-based reimbursement

Outcomes-based reimbursement has seen enormous development with the proliferation of pay-for-performance, value-based care, and affordable care organizations. CMS has led efforts to bring value-based care forward with Pay for Performance models and has increased access to EBPs through such mechanisms as Sect. 1115 waivers. These waivers allow states to test and implement approaches that support Medicaid program objectives that differ from what is allowed by federal statute [63]. Today, payment arrangements are rapidly moving away from volume-based payments (e.g., fee-for-service) towards value-based payments [63.] These payment models fall under three main umbrellas: (1) fee-for-service with enhanced payment for increased quality, (2) alternative payment models that utilize the architecture of the fee-for-service model with either shared savings, or shared savings and risk, and (3) population-based models which is the most evolved of these alternative payment approaches. The best example of the last, Accountable Care Organizations, or ACOs, is discussed in greater detail below.

#### Accountable care organizations

Earlier in this paper, we described what constitutes an ACO. In general, however, all newer payment models share the goals of managing soaring healthcare costs by eliminating duplicative services and reducing preventable hospitalizations and other complications of care. As alternative payment models evolve, there is increasing emphasis on moving towards population-based models that incentivize and remunerate healthcare providers for delivering high-quality, coordinated, person-centered care within a predetermined budget [64]. As of 2023, 14 states have reported ACOs [65]. Further, ACO contracts may support access to EBPs for people with persistent mental illness in some arrangements as provider organizations are incentivized by the potential cost savings [66, 67].

As of 2019, 46 states and 2 territories were implementing state-coordinated value-based reimbursement programs, leaving only Georgia, Louisiana, Mississippi, and Indiana with no coordinated value-based reimbursement strategy at the state level [63]. The past decades have solidified a shift from paying for processes to paying for outcomes, even as how to pay for outcomes is still being worked out.

### Changes in the political context

Since the original PEF, several changes have occurred in the political context that relate to the following areas: (1) EBP-focused legislation; (2) behavioral health parity laws; (3) COVID-19 mitigation efforts; (4) structural stigma; and (5) consumer involvement. We recognize that political and group-level factors within a state or county (e.g., the political party that controls the legislature) can be key to EBP support. For brevity, and in keeping with the theme of the paper, we discuss political actions that influence implementation, not the reasons for such actions.

#### EBP-focused legislation

Legislative strategies are blunt policy instruments. The PEF’s focus on legislation was based on the observation that policies that support access to, and quality of, health services ultimately wind up supporting implementation and sustainment of those services. An example of a policy promoting access to EBPs is the Family First Prevention Services Act of 2018 (FFPSA), which created the ‘Title IV-E Prevention Services Clearinghouse’ which contains a list of prevention services and programs that states can implement utilizing title IV-E funds [68]. Though the comprehensiveness of the clearinghouse is debated, this can increase the adoption of EBPs, and through such effects, support their widespread implementation [69]

One traditional way to increase access to EBPs is to legislate parity. The Mental Health Parity and Addiction Equity Act (MHPAEA) [70] and the ACA shared three overall goals—expand access to health insurance, improve coverage of mental health and substance use services, and extend the scope of coverage past medical-surgical benefits to include mental health and substance use benefits (MH/SUD) [71]. While MHPAEA codified significant new protections for consumers, sustained implementation of mental health and substance use services has not occurred as expected because of some problems with the design and implementation of the MHPAEA [72]. These problems include its complexity, including the involvement of enforcing agencies, and weak enforcement of parity [73]. MHPAEA’s rulemaking and enforcement provisions also shifted the onus onto the individual to file a complaint about non-compliance with the law [71, 74], such that non-implementation became an individual, rather than systemic, problem.

The enactment of the ACA on March 23, 2010, overcame some of MHPAEA’s shortcomings [37]. The ACA deemed mental health and substance use disorder services one of ten essential health benefits (EHB) and required non-grandfathered individual and small group plans to include these in coverage. The ACA lowered the estimated number of uninsured by approximately 20 million from 2010 to 2020 [75, 76], thereby laying the groundwork for scalability of services. The ACA also directly supported the implementation of preventative services, especially in states that chose to expand Medicaid eligibility [77]. The impact of the ACA today is seen in Medicaid expansions, and increased access to primary care. Through such demand-side expansions, access-focused pieces of legislation indirectly support large-scale implementation and create entitlements that can support the sustainment of specific EBPs and services.

#### Expanded reach in the context of COVID-19

The COVID-19 pandemic highlighted the tenuous link between ACA, MHPAEA, and the reality of seeking care. The Biden-Harris Administration—responding to reports of the impact of the pandemic on the nation’s mental health—highlighted alleviating the mental health crisis in the USA as a core aim of the Administration’s Unity Agenda. In response, several policies have passed to expand the reach of EBPs in the context of COVID, including insurance coverage of telehealth services [59].

#### Structural stigma

Structural stigma and discriminatory policies are important to consider as a key lever of the implementation of EBPs, especially in health and mental health settings [78, 79]. Corrigan and colleagues [80] define structural stigma in terms of “policies of private and governmental institutions that intentionally restrict the opportunities of people” and “policies of institutions that yield unintended consequences that hinder the options of people” (p. 481). Structural stigma relates to institutional racism, which captures the role of institutional, systemic, and cultural forces perpetuating racism against ethno-racially minoritized groups [81, 82]. Hatzenbuehler and Link [83] describe this as “societal level conditions, cultural norms, and institutional policies that constrain the opportunities, resources, and wellbeing of the stigmatized” (p. 2).

The past decades have recognized that stigma-focused policy strategies can enhance access and support the implementation of EBPs, especially for individuals and groups experiencing marginalization. The MHPAEA and the ACA both emphasize reducing structural stigma towards individuals with mental illness by improving access and coverage of services that were previously less accessible to them [71]. While less is known about the effects of structural stigma on the implementation of EBPs, Reid and colleagues [84] examined how structural stigma and discrimination can undermine the efficacy of psychosocial interventions. These findings capture how structural stigma and discrimination can serve as contextual moderator that can affect policy implementation. Identifying and dismantling such policies with disparitogenic effects related to structural stigma, discrimination, and exclusion may influence the *ecology* or *context* in which EBPs are implemented.

#### Consumer involvement

Service user participation in research, policy, and practice has increased in recent years [85]. Policies that support consumer involvement can improve the implementation of EBPs in mental health settings by creating a more inclusive landscape for delivering such services. Consumer involvement practices also enhance the equity and effectiveness of EBPs and create a more effective and humane service delivery system [85, 86]. The original PEF [7] argued that implementation efforts should actively build upon collaborative relationships with stakeholders as a lever to improve implementation and enhance the acceptability of many EBPs, and such efforts are well underway. A recent example is the Lancet Psychiatry’s Commission on Psychoses in Global Context [87]; this effort intends to increase the inclusion of individuals with lived experience in working groups and editorial boards to ensure that outputs and activities involve co-production. Another related effort to enhance the representation of consumers with lived experience is the development of grant review panels [88] including at the National Institute of Mental Health (NIMH) [89].

While participatory approaches to mental health services and implementation research have gained momentum, many of these efforts remain “surface level” such that stakeholder consultation is often limited to “one time” activities rather than genuine co-production [89, 90]. Initiatives that center service user involvement across all stages of the research process can be leveraged to enhance the dissemination and implementation of EBPs in mental health settings. Policy and organizational-level initiatives that genuinely increase service user participation are, therefore, critical from an implementation perspective. These initiatives have the potential to enhance the relevance of EBPs implemented in mental health settings.

### Changes in the social context

Since the original PEF, changes have occurred in the social context that primarily relate to social stigma.

#### Social stigma

The effects of mental health-related stigma–identified as an implementation challenge in the PEF–persist today, particularly among minoritized subgroups [82, 91, 92]. Stigma is a multifaceted construct that can be defined as a social process that involves:(1) labeling differences, (2) negative attributions and stereotypes, (3) distinguishing between “us” and “them,” and (4) experiences of discrimination and loss of status [82]. Stigmatization involves the labeling of “creditable” and “discreditable” identities that manifest through social processes and interactions [93].

A range of commissions and reports have suggested policy action to reduce stigma, including early examples such as the President’s New Freedom Commission of 2002, NIMH’s Stigma Working Group established in 1999, and more recently the National Academies of Sciences, Engineering, and Medicine’s report on ending stigma and discrimination against people with mental and substance use disorders [94–96]. These efforts suggest strategies such as supporting education, contact-based approaches, and public awareness campaigns [7]. Policy strategies that target mental health providers’ attitudes towards individuals with mental illness (“associative stigma”), e.g., through continuing education programs, and peer discussions, and supervision, are critical [97, 98]. Policies and legislation that support anti-stigma initiatives among the public, consumers, and providers can reduce *mechanistic barriers* to important mental health outcomes and help create an *ecology* and *context* that supports the implementation and uptake of EBPs in mental health settings.

## Future directions for policy levers to increase the reach of EBPs in mental health care

### Policymakers and their adoption decision-making

Although the PEF embraces an ecological approach and focuses on policy strategies at nested levels of context, it is important to recognize that the adoption of these strategies occurs through the decisions of *individual* policymakers. While these individuals typically make decisions collectively, the micro-level thoughts, feelings, and experiences of policymakers influence decisions to adopt and prioritize the enforcement of policy implementation strategies (as well as programs) [99–103]. Individually focused policymaker dissemination strategies have examined how to effectively communicate information about EBPs and policies, and such dissemination strategies could be adapted to enhance communication about the policy implementation strategies enumerated in the PEF. For example, dissemination strategies have been developed and tested to account for cognitive processes through which policymakers make decisions, their knowledge and attitudes about mental health issues, and mental models [104–106]. Decision support tools have been developed to inform policymakers’ individual decision-making process [107–109] and systems science methods—such as agent-based modeling—have been used to understand the dynamics through which policymakers’ make decisions with different agency contexts [110]. While this work has largely focused on policymaker decisions related to the adoption of specific programs and policies, it is applicable to fostering the uptake of policy strategies that would improve the implementation of EBPs in public systems.

### Policy ecology of sustainment

Policy efforts to sustain evidence-based interventions are in their nascency. In one national initiative in the USA to implement five EBPs across eight states, less than half of the initial programs showed sustained delivery of EBPs 6 years post-implementation [111]. In another county-wide effort to implement EBPs for children in Los Angeles, therapists sustained delivery of any given EBP for less than 2 years [112]. Sustainment is a contextual, and not just an organizational, attribute [113]. In other words, what to sustain and how to sustain it depends on service milieu, comparative advantage, and “moat” in addition to organizational capacity, leadership, and other intra-organizational strengths and competencies. Some of these determinants may be amenable to policy action, which is perhaps part of the reason behind the relative underdevelopment of policy strategies to support the implementation of best practices. Consequently, one future task will be to systematically identify policy-mutable targets of implementation sustainment, identify which tools or strategies in the PEF best engage those targets, and test the effects of those tools on the sustainment of best practices in the short and long run.

### Policy strategies to support de-implementation

Although the PEF was developed to characterize how policy can support the implementation of EBPs, the framework could also inform policy strategies to support the *de-implementation* of ineffective, harmful, or inequitable programs. While program de-implementation has received some substantive attention [114–117] limited research has focused on the role of policy in fostering de-implementation [46]. Across the four domains of the PEF, there are ways in which policy strategies could inadvertently promote the sustainment of interventions that should be de-implemented. One could also imagine ways in which the strategies could be “reverse engineered” to actively facilitate the de-implementation of programs. In the domains of political and agency context, there may be a need for dissemination strategies that increase knowledge about programs that should be de-implemented and cultivate will for de-implementation among policymakers and organization leaders. A 2020 survey of substance use agency policymakers found that de-implementation of non-evidence-based interventions was rated as the lowest priority out of 14 issues [118].

### Evidence for policy

That policymakers do not eagerly embrace policy-directed research is an old lament. In more recent writings, the nature of evidence that policymakers seek has been subject to continued investigation. Brian Head, for example, outlines the sometimes “data-resistant” nature of political decision-making, the “three lenses” that policymakers tend to use while evaluating evidence, of which scientific evidence is just one such lens, and the networked and shared governance models that empower different kinds of evidence [119]. The key insight is that there is no single evidence base for policy decisions; instead, we need to think about evidence *bases*. Strengthening the scientific evidence base, and outlining the kinds of research needs that can do so, is a critical first step in this broader process, as outlined in a recent work on the nature of evidence for implementation science [120]. Consequently, a policy-focused implementation framework can certainly highlight tools available to policymakers but should recognize that as the context and evidence behind these tools shift, these tools will also shift [5].

## Summary

This iteration of the PEF expands and enhances the specification of available policy levers and targets that may provide opportunities for policy-level support. While some legislative strategies have delivered mixed results, policy action remains a key tool for implementation efforts nationwide. Furthermore, the focus on increasing the public health impact of EBPs remains steady. The updated PEF clarifies current policy efforts within the field of implementation science in health to conceptualize and de-mystify the role of policy in the implementation of EBPs.

### Supplementary Information

**Additional file 1. **Developing the Policy Ecology Framework (2008) and Looking Forward.

## Data Availability

Not applicable.

## Abbreviations

ACA: Patient Protection and Affordable Care Act
ACO: Accountable Care Organization
CCBHCs: Certified Community Behavioral Health Clinics
CMS: Centers for Medicare and Medicaid Services
DOL: Department of Labor
EBP: Evidence-based practices
EPIS: Exploration, Preparation, Implementation, Sustainment
HHS: Department of Health and Human Services
MHPAEA: Mental Health Parity and Addiction Equity Act of 2008
NIMH: National Institute of Mental Health
PEF: Policy Ecology Framework
PA: Prior authorization
USA: United States of America
