# Supplementary material for: Revisiting the policy ecology framework for implementation of evidence-based practices in mental health settings
Source: Implement Sci. 2023 Nov 7;18:58. doi: 10.1186/s13012-023-01309-9 (PMC10629012; doi:10.1186/s13012-023-01309-9)
Supplement: Supplementary file 1 — Additional file 1. Developing the Policy Ecology Framework (2008) and Looking Forward. [file 13012_2023_1309_MOESM1_ESM.docx]

| **Additional File 1. Developing the Policy Ecology Framework (2008) and Looking Forward** | | |
| --- | --- | --- |
|  | **Activity** | **Process** |
| **Step 1: Origins and Conceptualization of the PEF** | - Originated within the Policy Core of the National Child Traumatic Stress Network (NCTSN). - Conceptualized as a functional toolkit to support policy action in response to efforts to improve child mental health care. | - Employ an ecological framework adapted for EBP implementation. - Identified and organized policy levers (strategies) at the organizational, regulatory, political, and social levels that support sustainable uptake. |
| **Step 2: Integration of the PEF into Implementation Science Literature** | - Reviewed applications of the PEF in three major system-improvement efforts in Philadelphia, Maryland, and Minnesota. | - Authors reviewed applications of the PEF to gain insight into how the framework has been utilized on the ground. - Authors reviewed articles citing the original PEF (n=180) to examine how the PEF was applied. |
| **Step 3: Collection of Targeted Evidence to Inform the Update of the PEF** | - Reviewed federal legislation and accompanying set of policy levers. - Reviewed international interest and deployment of policy strategies that drive health system reform efforts. | - Determined objectives for the update: (1) map changes in the policy landscape, (2) mark the further development of existing policy levers, (3) identify novel policy levers that support evidence-based interventions, and (4) highlight examples of the deployment of these policy strategies. - Determined the manuscript to be formatted as a debate; therefore, no systematic review was pursued. - Searched bibliographic databases: (Scopus, PsycNET (with PsycINFO), ProQuest Central, Web of Science). APA PsychINFO, govinfo, and PEW Trusts were searched for grey literature. Search terms used were targeted to each subsection. - Quantitative, qualitative, mixed-method studies, review papers, reports, and conceptual papers were included to consider different aspects of implementation policy. |
| **Step 4: Adaptation of the PEF** | - Updated the ecology to reflect changes in the policy landscape in order to provide accurate guidance to policymakers aiming for sustainable implementation of EBPs. | - Key findings were collated through a narrative synthesis approach. Authors grouped the papers by PEF domain, presented the status of the policy action, and summarized key changes that have occurred since the publication of the original PEF. - Dashed lines in the ecology as the boundaries in policy ecology are not clearly defined in reality (Figure 1). |
| **Step 5: Expansion of the PEF** | - Outlined some of the future directions or applications of the strategies or “tools” that the PEF identified. | - Reviewed need for policy-mutable targets and provided an overview of the PEF in relation to adoption decision-making, sustainment, and de-implementation. |
